# Short-term cactus pear [*Opuntia ficus-indica* (L.) Mill] fruit supplementation ameliorates the inflammatory profile and is associated with improved antioxidant status among healthy humans

**DOI:** 10.29219/fnr.v62.1262

**Published:** 2018-08-20

**Authors:** Alessandro Attanzio, Luisa Tesoriere, Sonya Vasto, Anna Maria Pintaudi, Maria A. Livrea, Mario Allegra

**Affiliations:** Dipartimento di Scienze e Tecnologie Biologiche, Chimiche e Farmaceutiche, Università degli Studi di Palermo, Palermo, Italy

**Keywords:** cactus pear fruit, healthy subjects, inflammatory biomarkers, antioxidant network, skin carotenoids

## Abstract

**Background:**

Dietary ingredients and food components are major modifiable factors protecting immune system and preventing the progression of a low-grade chronic inflammation responsible for age-related diseases.

**Objective:**

Our study explored whether cactus pear (*Opuntia ficus-indica*, *Surfarina* cultivar) fruit supplementation modulates plasma inflammatory biomarkers in healthy adults. Correlations between inflammatory parameters and antioxidant status were also assessed in parallel.

**Design:**

In a randomised, 2-period (2 weeks/period), crossover, controlled-feeding study, conducted in 28 healthy volunteers [mean age 39.96 (±9.15) years, BMI 23.1 (±1.5) kg/m^2^], the effects of a diet supplemented with cactus pear fruit pulp (200 g, twice a day) were compared with those of an equivalent diet with isocaloric fresh fruit substitution.

**Results:**

With respect to control, cactus pear diet decreased ( *p* < 0.05) the pro-inflammatory markers such as tumour necrosis factor-α (TNF-α), interleukin (IL)-1β, interferon-γ (INF)-γ, IL-8, C-reactive protein (CRP) and erythrocyte sedimentation rate (ESR), whereas it increased ( *p* < 0.05) the anti-inflammatory marker IL-10. Moreover, the diet decreased ratios between pro-inflammatory biomarkers (CRP, IL-6, IL-8, TNF-α) and anti-inflammatory biomarker (IL-10) ( *p* < 0.05). Cactus pear supplementation caused an increase ( *p* < 0.05) in dermal carotenoids (skin carotenoid score, SCS), a biomarker of the body antioxidant status, with correlations between SCS and CRP (*r* = −0.905, *p* < 0.0001), IL-8 (*r* = −0.835, *p* < 0.0001) and IL-10 (*r* = 0.889, *p* < 0.0001).

**Conclusions:**

The presently observed modulation of both inflammatory markers and antioxidant balance suggests cactus pear fruit as a novel and beneficial component to be incorporated into current healthy dietary habits.

Dietary patterns based on plant-derived food have consistently been associated with reduced risk of age-related pathologies, such as cardiovascular diseases, cancer, metabolic syndrome, inflammatory conditions and neurodegenerative disorders (1). A slowly increasing systemic inflammation is related to the onset and the evolution of virtually all the above-mentioned pathologies. Indeed, serum concentrations of inflammatory mediators typically increase with age even in the absence of acute infection or other pathological stress. In this context in order to examine the ‘diet-health’ relationship, dietary interventions targeting disease prevention are critically dependent on the evaluation of inflammatory biomarkers, even within the reference range, to monitor underlying inflammatory state and progression. Notwithstanding the complexity and the discordance of the available literature in this regard, a suite of biomarkers reliable even in low-grade inflammation has emerged in the recent years (2).

Inflammation-induced tissue damage occurs in an organ-specific manner in different diseases or conditions; nonetheless, responses and markers in different organs and molecules involved in the inflammatory reaction are remarkably similar. Along with an increased number of peripheral leukocytes, an overproduction of inflammatory cytokines and chemokines, including tumour necrosis factor-α (TNF-α), interleukin (IL)-1β, IL-6, IL-8 and interferon-γ (INF-γ), is observed (3, 4). These mediators act to amplify the inflammatory process and elicit systemic effects, such as generation of high amounts of hepatic acute-phase reactant C-reactive protein (CRP), eventually leading to tissue injury. All these parameters have then been identified as a set of ‘general’ biomarkers that can provide quantitative assessments of the inflammatory condition [2]. On the contrary, the concentration of anti-inflammatory cytokine IL-10 (5, 6) and its relationship and balance with the pro-inflammatory ones has to be taken into consideration when evaluating ongoing inflammatory processes and specifically age-related low-grade inflammation (7).

Within the abundance of fruits and vegetables, the *Opuntia ficus-indica* fruit has received a reasonable amount of attention over the last decade for its health-promoting properties mainly related to its betalain content (8–17). In particular, human studies showed that consumption of cactus pear at dietary-achievable amounts was associated with a remarkable reduction of oxidative stress in healthy subjects (18). Moreover, nutritionally relevant supplementation of either whole fruit extracts or its characteristic betalain and indicaxanthin has been reported to exert strong anti-inflammatory effects in several experimental models both *in vitro* and *in vivo* (11–15, 17).

Inflammatory biomarkers undergo an age-related increase, though remaining within a normal range, in healthy subjects (7). In this context, the primary objective of this short-term dietary intervention study was to investigate whether dietary amounts of cactus pear fruit, daily supplemented for 2 weeks, could affect inflammatory status and plasma concentration of inflammatory biomarkers of healthy men and women. Currently, no single marker can predict the effect of a dietary intervention, and very few studies have considered multiple markers simultaneously, most focusing on non-specific biochemical markers such as CRP and/or IL-6 (19–23). Conversely, in our study both clinical biomarkers, such as CRP and erythrocyte sedimentation rate (ESR), and a large panel of pro- and anti-inflammatory cytokines have been considered. Moreover, owing to the interconnections between inflammation and oxidative stress (24), we concurrently assessed the effects from cactus pear supplementation on the global body antioxidant balance and sought to correlate them with the anti-inflammatory ones.

## Subjects and methods

### Fruit

Cactus pear fruits from the yellow Sicilian *Surfarina* cultivar, from the same area (San Cono, Catania, Italy), at comparable ripening stages and 1-week old, were obtained from the producer from October to December 2016, and the study was carried out within the same time interval. The necessary amounts were delivered to our laboratory twice a week. Fruits were peeled and pulp consumed according to the design below. The phytochemical content of the fruit was randomly checked at the beginning of the study and once a week during the time of supplementation according to methods reported elsewhere (8, 25).

### Participants, study design and compliance

The study population consisted of 28 volunteers aged 30–69 years (14 women, 14 men; age 31–40 years, *n* = 15; 40–50 years, *n* = 10; 50–60 years, *n* = 2; >60 years, *n* = 1; *x* ± SD = 39.96 ± 9.15); body mass index (kg/m^2^): 23.1 ± 1.5. All participants were highly educated, working at our university (*n* = 12), professionals (*n* = 6) or postdoc students (*n* = 10). Among them, some worked in the field of health science (*n* = 13). Some participants performed habitual physical activity (*n* = 4), and some were smokers (*n* = 6). They provided written informed consent to participate and none of them withdrew from the study. All participants were in good general health as determined by a medical history questionnaire, physical examination and clinical laboratory tests. Subjects fulfilled the following eligibility criteria: (1) no history of cardiovascular, hepatic, gastrointestinal or renal disease, hypertension, diabetes mellitus and dyslipidaemia; (2) no antibiotic, vitamin or mineral use for > 6 weeks before the beginning of the study. The women were not using exogenous hormones. The study protocol was in accordance with the Helsinki Declaration of 1964 and its later amendments.

**Fig. 1:**
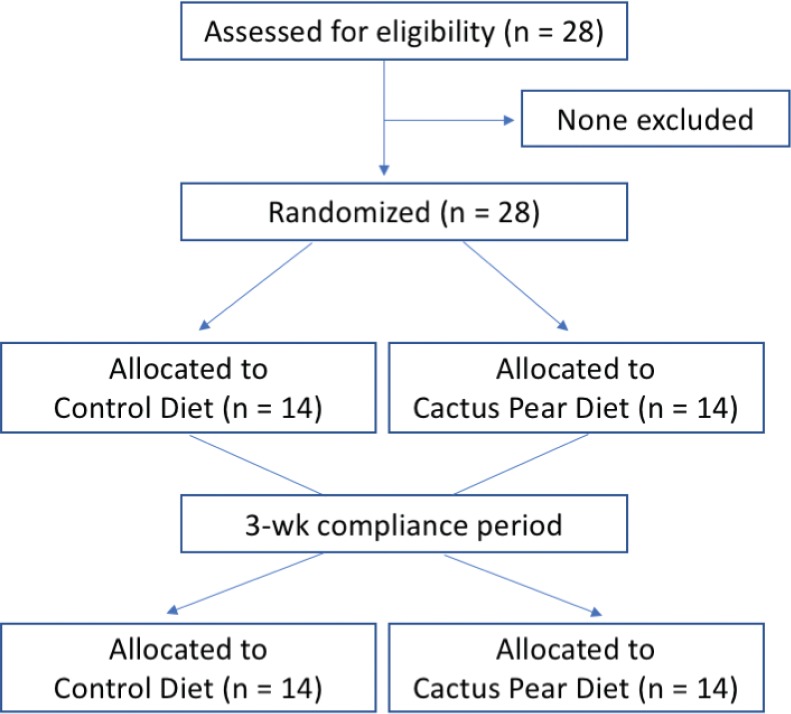
Scheme of participant flow through the study.

The study was a randomised, crossover, 2-period (2 weeks/period), controlled-feeding study designed to evaluate the effects of cactus pear fruit pulp consumption (200 g, twice a day) in the context of a planned dietary regimen, on both a number of inflammatory parameters and the whole body antioxidative status. Blood samples, collected in EDTA-coated tubes (1 mg/mL) after an overnight fasting, were obtained from the subjects at the beginning (baseline, B) and at the end of each diet period, that is, control diet (CD) and cactus pear diet (CPD). Diet periods were separated by a 3-week compliance break after which blood was drawn. Plasma was separated from blood cells by centrifugation at 4°C and 2,000 × *g* for 10 min. Suitable aliquots of plasma were portioned, stored at −80°C and analysed within 2 weeks. The trial design is depicted in Fig. 1. With the exception of the substitution of seasonal fruit with cactus pear fruits, participants consumed individual but comparable meals. Energy, macronutrient, total lipids, cholesterol, saturated fatty acids and vitamins were calculated by WinFood Software based on the Italian Food Composition Table from the National Institute of Nutrition (INRAN). The individual dietary plans were composed using information from each subject with the objective of respecting the personal food tastes and according to the total energy expenditure for each participant. Macronutrient distribution was adjusted in accordance with the Acceptable Macronutrient Distribution Range (26). Participants committed themselves to substitute equivalent foods using a food replacement list based on MyPlate’s food groups, as vegetables, grains, protein, dairy and others (oil, sugar and salt) (27). Food portion size was based on Italian standard kitchen utensils, and all participants were recommended by the dietician to use them to measure their own consumption. Dietary composition reported in Table 1 was based on the planned dietary protocols and consisted of five meals daily, that is, *breakfast*: milk or yogurt, coffee, whole-grain bread with jam; *snack 1*: 200 g of seasonal fresh fruit (CD, Table 2) or 200 g of cactus pear fruit pulp (CPD); *lunch*: pasta or bread or rice, grilled fish, salad or cooked vegetables; *snack 2*: 200 g of seasonal fresh fruits (CD, Table 2) or 200 g cactus pear fruit pulp (CPD); *dinner*: bread, legumes or grilled fish, cooked vegetables or salad. Food intake was assessed using 3-day food records, applied four times for each patient (1 week, 3 weeks, 5 weeks and 7 weeks). All raw dietary components were provided to participants twice a week, and the subjects were instructed for the meal preparation by written recipes. Record methodology, portion size estimate and validation were conducted in agreement with published protocols (27). Participants were recommended to maintain their lifestyle habits.

**Table 1:** Macro- and micro-nutrient composition of cactus pear diet (CPD) and control diet (CD), on the basis of 2100 kcal/day and averaged across a 5-day menu cycle.

	CPD	CD
Protein, % of kcal (g)	13.5 (71)	14.3 (75)
Carbohydrate, % of kcal (g)	59.8 (314)	58.7 (308)
Fat, % of kcal (g)	26.6 (62)	27.0 (63)
SFA, % of kcal (g)	7.4 (18)	7.6 (18)
MUFA, % of kcal (g)	10.1 (24)	10.3 (24)
PUFA, % of kcal (g)	6.1 (15)	6.3 (15)
Cholesterol, mg	125	120
Fiber, g	24.1	25.1
Sodium, mg	3012	3018
Potassium, mg	2860	2855
Calcium, mg	1329	1331
Iron, mg	14	16
Ascorbic acid, mg	130	120

Note: All values were determined using. The Food Processor SQL (version 10.8.0; ESHA Research, Salem, OR). MUFA indicates monounsaturated fatty acids; PUFA, polyunsaturated fatty acids; SFA, saturated fatty acids.

**Table 2:** Types of fruit consumed in the CD.

Snack 1	Strawberry
	Pineapple
	Apple
	Tangerine
Snack 2	Orange
	Melon
	Pear
	Grapefruit

### Assessment of hematologic indexes and inflammatory markers

Glucose was measured using the hexokinase method; total cholesterol, high-density lipoprotein (HDL) cholesterol and triglycerides were measured using enzymatic colorimetric methods (Abbott ARCHITECT System; Abbott Diagnostics, Abbott Park, Ill). Low-density lipoprotein (LDL) cholesterol was calculated using the Friedewald formula.

Serum concentrations of all plasma cytokines were measured by high-sensitivity magnetic luminex performance assay (R&D Systems, Minneapolis, MN) in triplicate. Serum high-sensitivity CRP was measured by latex-enhanced immuno-nephelometry (Quest Diagnostics, Madison, NJ). ESR was determined as reported by Strojnik et al. (28).

### Measurement of skin carotenoids as index of body antioxidant status

A portable Raman spectroscope, Pharmanex^®^ Bio-Photonic Scanner S2 (NuSkin, Provo, Utah, USA), designed to monitor carotenoids in the 0.1 mm stratum corneum of the skin of the hand, has been used for the measurement of the global antioxidant status (29). A low-intensity 471.3–473 nm radiation from light emitting diodes interacts with the skin carotenoids. The scattered light is detected at 507.8–509.8 nm by the scanner that converts the Raman intensity in counts (skin carotenoid score, SCS). A computer then transforms the scanner signals in a coloured scale going from red (poor SCS, <19,000) to dark blue (high SCS, >50,000). SCS can be converted to laboratory measurements using the equation [Y = 12703X + 5891.7], where ‘Y’ is the SCS value and ‘X’ is the carotenoid concentration expressed as micrograms (μg)/mL of serum.

### Statistical analysis

Data are expressed as means ± SD. Comparisons were made using one-way ANOVA followed by Sidak’s multiple comparison test. In all cases, significance was accepted when the null hypothesis was rejected at *p* < 0.05 level. Pearson’s correlational analysis was run on comparisons of IL-10, IL-8 and CRP versus SCS.

## Results

### Compliance of subjects to the study

The compliance of each patient to his/her dietary schedule was assessed by nutritionists every week during the intervention and was very good as no one dropped out of the study or reported problems in the self-management of meals; no adverse events were identified in both CD and CPD over the study period, and all 28 subjects (14 in the CD group and 14 in the CPD group) completed the study and were included in the analyses (Fig. 1).

### Phytochemical profile of cactus pear fruits

The fruit content was checked for antioxidant vitamins, betalain pigments and polyphenols and results are shown in Table 3. Significant amounts of ascorbic acid and indicaxanthin and minor amounts of β-carotene were measured, whereas polyphenols were almost absent, with the exception of minor amounts of kaempferol.

**Table 3:** Phytochemicals, vitamins, antioxidants and total reducing power (TEAC) of the cactus pear pulp employed in the study.

	Content/100 g edible pulp
Indicaxanthin (mg)	8.51 ± 0.33
Betanin (mg)	1.05 ± 0.19
Ascorbic acid (mg)	29 ± 2
α-Tocopherol (μg)	75 ± 6
β-Carotene (μg)	1.3 ± 0.5
Glutathione (mg)	7.8 ± 0.5
Cysteine (mg)	0.67 ± 0.04
Kaempferol (μg)	2.1 ± 0.3

Note: All values are the mean ± SD of five determinations performed in triplicate on four lots of fruits.

### Effect of cactus pear supplementation on CRP and ESR

Characteristics of the participants to the study are summarised in Table 4. All parameters measured were within the reference range at the beginning of the study (baseline), and their level did not vary significantly at the end of neither the control diet nor the cactus pear diet.

**Table 4:** Lipid haematological parameters of the subjects enrolled at baseline, after CPD and after CD.

	Baseline	CPD	CD
Glucose (mmol/L)	4.60 ± 0.41	4.38 ± 0.39	4.94 ± 0.42
Cholesterol (mmol/L)	4.84 ± 0.42	4.69 ± 0.44	4.94 ± 0.39
HDL-cholesterol (mmol/L)	2.56 ± 0.2	2.46 ± 0.1	2.61 ± 0.2
LDL-cholesterol (mmol/L)	2.28 ± 0.2	2.23 ± 0.2	2.33 ± 0.2
Triglycerides (mmol/L)	1.12 ± 0.09	1.07 ± 0.09	1.14 ± 0.1

Note: All values are the mean ± SD of separate determinations performed in triplicate on blood samples from different subjects, *n*=28. With respect to baseline, no value was significantly different. One-way ANOVA with Sidak’s multiple comparison test.

**Fig. 2:**
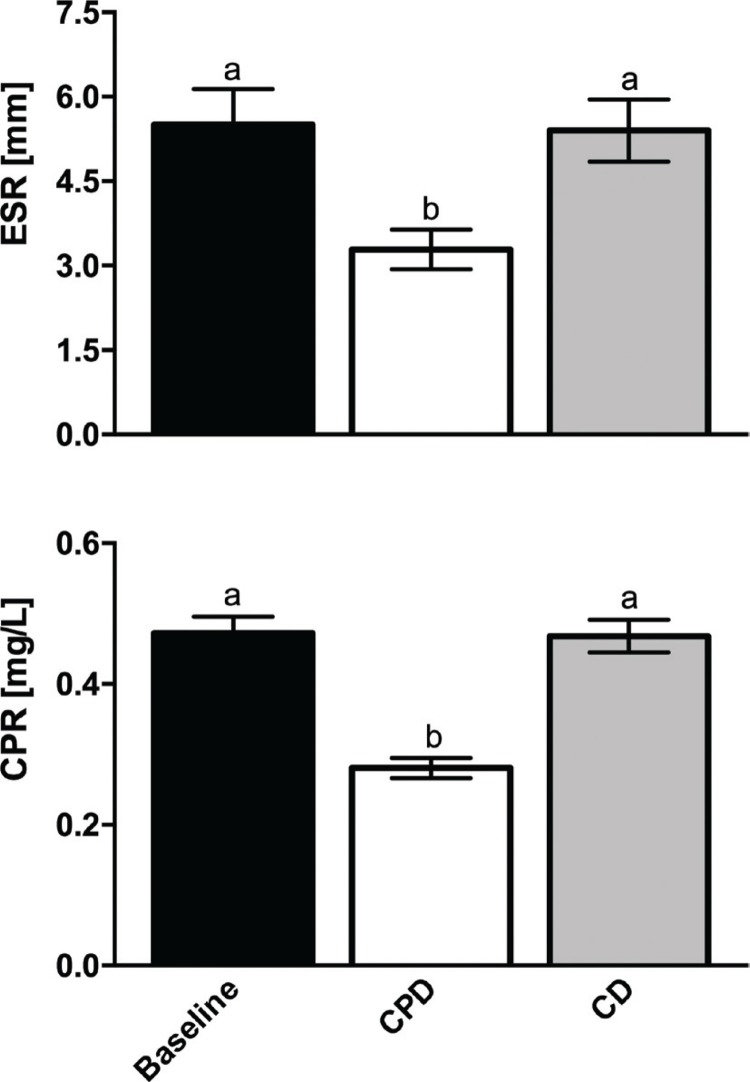
Plasma ESR and CRP values of the subjects enrolled at baseline, after CPD and after CD. All values are the mean ± SD of separate determinations performed in triplicate on samples from different subjects, n = 28. Labelled means without a common letter differ with a *p* < 0.05. One-way ANOVA with Sidak’s multiple comparison test.

Levels of CRP and ESR were measured as non-specific markers of the global inflammatory status. CRP and ESR should not exceed 10 mg/L and 15 mm/h, respectively, in healthy subjects (30). In our study, both values were in the reference range at the baseline (CRP = 0.47 ± 0.12; ESR = 5.5 ± 2.38) and were not significantly modified by CD; however, they showed a significant ( *p* < 0.02 for CRP and *p* < 0.0001 for ESR) decrease after the cactus pear supplementation (Fig. 2).

Level of CRP and ESR evaluated at the end of the 3-week compliance period was not significantly different from baseline values (not reported).

### Effect of cactus pear supplementation on inflammatory markers

**Fig. 3:**
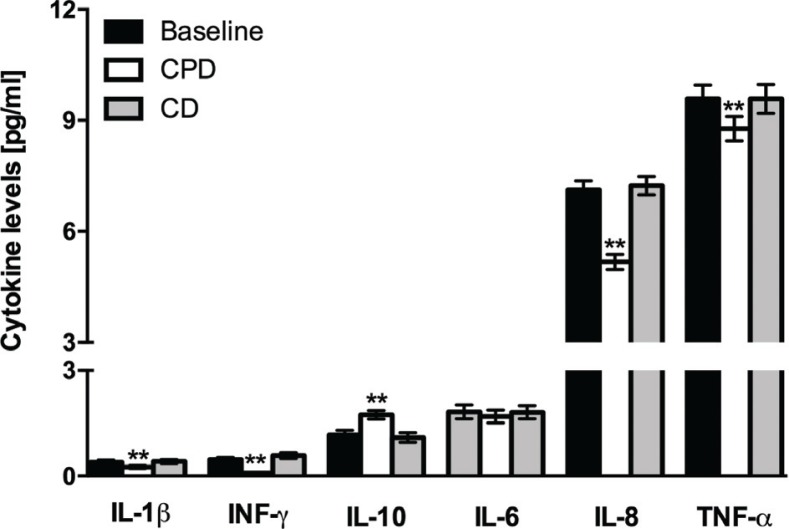
Plasma cytokine levels of the subjects enrolled at baseline, after CPD and after CD. All values are the mean ± SD of separate determinations performed in triplicate on samples from different subjects, *n* = 28. Within each set, asterisks indicate that means differ with a *p* < 0.05. One-way ANOVA with Sidak’s multiple comparison test.

Taking into account the strong relationship between CRP and cytokine production (31, 32), we next evaluated the effect of cactus pear fruit supplementation on several plasma cytokines. With respect to CD, almost all cytokines tested varied significantly after CPD ( *p* < 0.05) (Fig.3). Noteworthy, while remaining within a physiological range, levels of pro-inflammatory cytokines (i.e. TNF-α, IL-1β, INF-γ and IL-8) decreased, whereas that of IL-10, a potent anti-inflammatory cytokine, increased. IL-6 did not show any significant variation (Fig. 3).

The level of cytokines at the end of the 3-week compliance period was not significantly different from the baseline level (not reported).

Ratios between pro- and anti-inflammatory markers can help to define asymptomatic low-grade inflammation (33). Table 5 reports the calculated ratios between CRP, IL-6, IL-8, TNF-α and IL-10 at baseline and at the end of CPD and CD. Although serum concentration of IL-6 did not vary at any dietary condition, a significant decrease ( *p* < 0.0002) in IL-6/IL-10 ratios was observed after CPD as compared with CD. Likewise, there was a decrease of all other pro-inflammatory mediators/IL-10 ratios ( *p* <0.0001).

**Table 5:** Ratio between selected inflammatory parameter (IL-6, IL-8, TNF-α, CRP) and IL-10 at baseline, after CPD and after CD.

	Baseline	CPD	CD
IL-6/IL-10	1.84 ± 0.98	1.02 ± 0.52*	2.05 ± 1.18
IL-8/IL-10	7.36 ± 3.03	3.26 ± 1.26**	8.35 ± 3.95
TNF-α/IL-10	10.30 ± 5.10	5.55 ± 2.14**	11.60 ± 6.80
CRP/IL-10	0.46 ± 0.27	0.17 ± 0.10**	0.49 ± 0.31

Note: All values are the mean ± SD of separate determinations performed in triplicate on blood samples from different subjects, *n* = 28. With respect to baseline, values significantly differ (*) with *p* = 0.0002, (**) with *p* < 0.0001. One-way ANOVA with Sidak’s multiple comparison test.

### Effect of cactus pear supplementation on antioxidant state

**Fig. 4:**
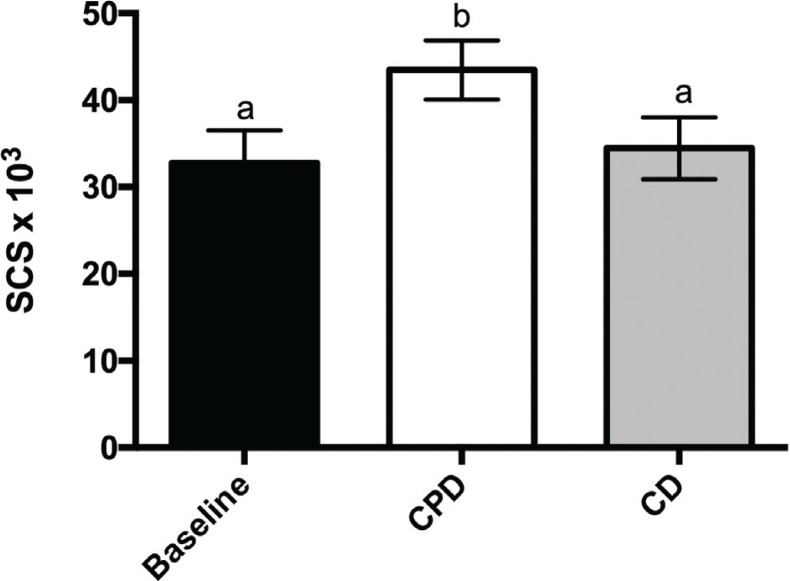
SCS values of the subjects enrolled at baseline, after CPD and after CD. All values are the mean ± SD of separate determinations performed in triplicate on samples from different subjects, *n* = 28. Labelled means without a common letter differ with a *p* < 0.05. One-way ANOVA with Sidak’s multiple comparison test.

**Fig. 5:**
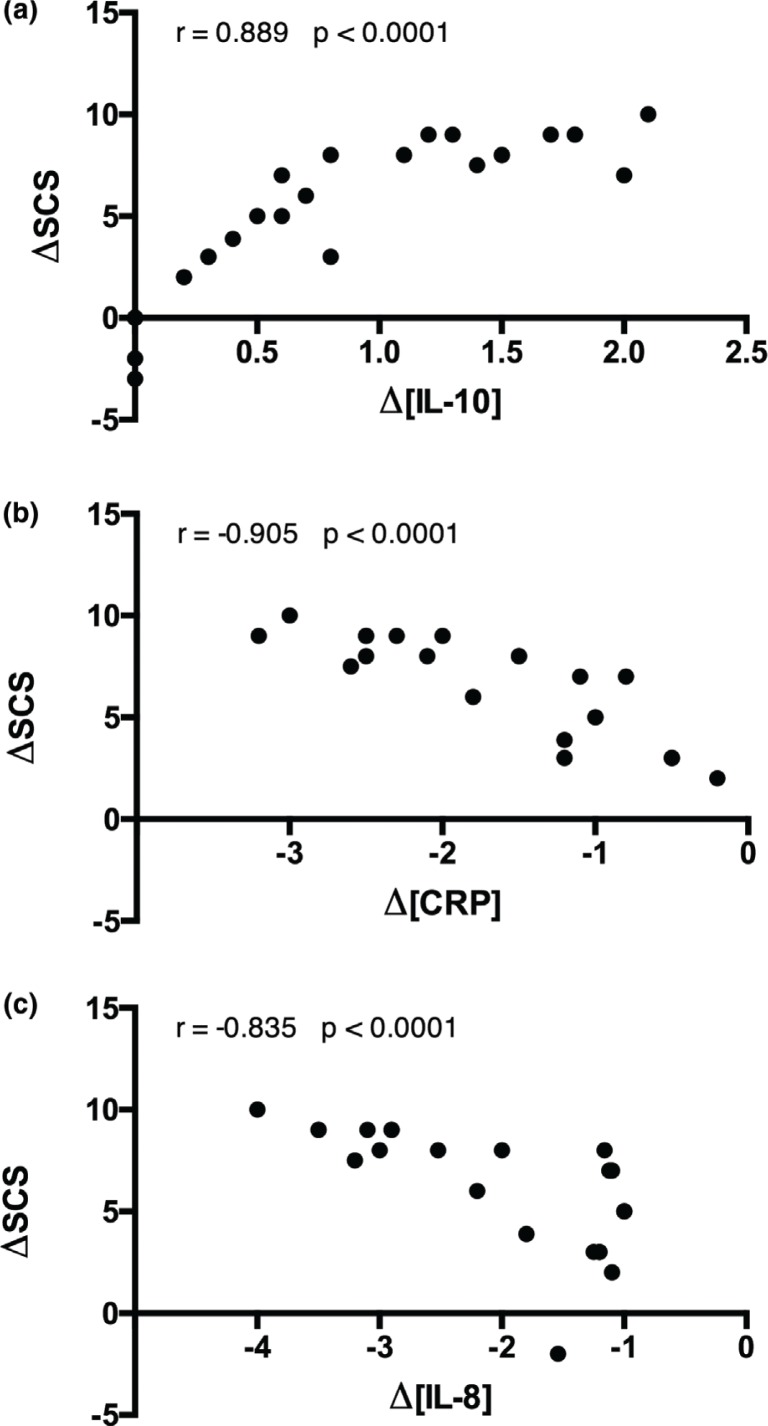
Correlation between changes of SCS and IL-10 (a), CRP (b) and IL-8 (c) values of the subjects enrolled at baseline and after CPD. All values are the mean ± SD of separate determinations performed in triplicate on samples from different subjects, *n* = 28. The Pearson’s correlation coefficients and *p*-values are stated in the corresponding plots.

The antioxidant status of the subjects was assessed through Raman spectroscopy-based measurements of skin carotenoids. At the baseline, SCS was 30 × 10^3^ to 40 × 10^3^ (Fig. 4). In comparison with CD, CPD significantly ( *p* < 0.05) improved the SCS (40 × 10^3^ to 50 × 10^3^). Remarkably, a negative correlation existed at the end of CPD between the change of the skin carotenoid levels and either CRP or IL-8, whereas a positive correlation was observed with the anti-inflammatory IL-10 (Fig. 5). On the contrary, no significant correlation was found between the change of the SCS and the change of ESR, TNF-α, IL-1β and TNF-γ.

## Discussion

Monitoring the inflammatory status of healthy subjects following specific dietary approaches may help to reveal food components that may protect the immune system and counteract its progressive dysfunction. By comparing the effects of a cactus pear fruit including diet with those of a comparable diet with a seasonal fresh fruit substitution, this study showed that a 2-week supplementation with dietary amounts of cactus pear fruit significantly modulated a large panel of critical inflammatory biomarkers, this being correlated with concurrent amelioration of the body antioxidant status, in healthy adults.

Serum CRP and ESR are general but definite inflammatory biomarkers; in line with current literature data showing that consumption of healthy dietary patterns rich in vegetables, fruits and legumes is associated with a significant reduction of both CRP and ESR (1, 34), we here observed a remarkable decrease of both these parameters after cactus pear supplementation. In addition, beside its significance in the inflammatory reaction, CRP is considered among risk factors for CVD and independent predictor of cardiovascular events (35). The reported significant reduction of plasma CRP by cactus pear fruit supplementation may suggest further benefits from diets enriched with these fruits.

The effects from cactus pear supplementation on CRP levels were paralleled by those on several plasma cytokines, with the exception of IL-6. The latter plays an important role in the inflammatory response and is a key stimulus for the acute-phase response that drives downstream events, including the hepatic synthesis of CRP (36). For this reason, a positive correlation between levels of IL-6 and CRP usually exists under inflammatory conditions. As stated by our measurements, despite the remarkable decrease in CRP, IL-6 was not affected by the supplementation. Dietary components may possibly affect the levels of CRP in different ways in healthy subjects. Liver pathways regulating CRP production are indeed controlled by a number of pro- and anti-inflammatory factors, including IL-10 (33, 37, 38)*.* The increase in this anti-inflammatory cytokine, caused by cactus pear supplementation, may have altered the cytokine balance eventually preventing the activation of the CRP synthesis.

Besides the serum levels of inflammatory mediators, ratios between pro- and anti-inflammatory markers can help to define asymptomatic low-grade inflammation (33) and should therefore be considered in healthy subjects. We observed a significant decrease in the ratio between CRP, IL-6, IL-8, TNF-α, all factors that may undergo a life-long variation within their normal range in healthy population (7), and IL-10. Although serum concentration of IL-6 did not vary, the decrease in IL-6/IL-10 ratio further indicates the positive effect of cactus pear on the inflammatory status of the subjects and confirms a prevalence of activation of anti-inflammatory pathways. Then, cactus pear fruit supplementation might have, by some means, affected physiological mechanisms of control, in turn causing a decrease in pro-inflammatory factors and an increase in anti-inflammatory factor.

Anti-inflammatory effects arising from dietary fruits and vegetables have possibly been related to the flavonoid content (34). Interestingly, only minor amounts of kaempferol occurred in our cactus pear fruits. A modulation of redox-dependent inflammatory pathways by indicaxanthin (13), the main and highly bioavailable (39) phytochemical of this yellow cultivar of cactus pear fruit that was used, may be implicated. In addition, although we demonstrated that vitamin C was not involved in the beneficial effects by cactus pear supplementation on the oxidative stress (18), present findings cannot rule out a positive role for ascorbate or for a combination of betalain pigments with ascorbate on the inflammatory status.

The global body’s antioxidant status is a result of a balance between the level of antioxidants in cells and body fluids, including blood, and pro-oxidant species endogenously produced or coming from external sources. Body’s molecular antioxidants work in concert and preserve each other, so the level of each one in the pool reflects the level of the whole antioxidant pool. On this basis, dermal carotenoids are a feasible marker of the body antioxidative network and may provide a useful, though indirect, indication of a moderate to severe individual oxidative stress (40). By a non-invasive laser spectroscopy-based measurement of skin carotenoids, we showed that cactus pear consumption caused an increase of skin carotenoids by around 30%, showing that the antioxidant balance of the subjects varied positively. Data from the present approach are substantially in accordance with those from blood measurements previously reported by our research group, showing reduction of oxidative stress arising in healthy subjects from cactus pear fruit consumption (18). It is not realistic to suppose that the very minute amounts of β-carotene in the fruits might vary the blood amounts of β-carotene (of the order of 10^−6^ M in healthy people), and then the skin content, to the observed extent. Rather, we suggest that the ingestion of the whole fruit and its phytochemical components, including betalains, can favourably affect cell pathways concurring to the maintenance of a proper antioxidant status (41). Moreover, in accordance with the interconnections between inflammation and oxidative stress (24), we observed that the antioxidative effects from cactus pear ingestion had a meaningful correlation with the anti-inflammatory factor. Specifically, the increase in the changes of SCS negatively correlated with the changes of CRP and IL-8 and positively with those of the anti-inflammatory IL-10.

A limitation of the study is the rather small sample size including young- to middle-aged healthy subjects from the same area. A second limitation is the short duration of the intervention. Nonetheless, the experimental design is quite coherent with previous nutritional investigations from this group (18), showing reduction of oxidative stress in healthy volunteers by regular consumption of cactus pear fruits. Exploring the effect of cactus pear fruit supplementation on the immune system in different age groups, as well as subjects at risk or affected by inflammatory diseases, should deserve further studies.

## Conclusive remarks and perspectives

Dietary ingredients and food components are major modifiable factors affecting immune function and may lifelong counteract ongoing alterations of the inflammatory state associated with ageing and age-related disorders (33, 42). In this context, our randomised, crossover, controlled-feeding study in healthy men and women provides a good indication of the potential of this fruit to positively affect mechanisms regulating the immune system. Moreover, by providing evidence that relationships between the circulating levels of inflammatory biomarkers and the individual oxidative status may be revealed in healthy humans, our findings may suggest an interesting issue for nutritional investigations. Since inflammation and oxidative stress contribute to the pathogenesis of many of chronic pathologies, assessment of their relationships in healthy populations may help to predict risk of age-related chronic conditions and eventually validate the effects of dietary interventions.

Present findings may suggest cactus pear fruit as a novel habit to be incorporated into the dietary portion of beneficial lifestyle changes.
